# Quantitative discrimination between invasive ductal carcinomas and benign lesions based on semi-automatic analysis of time intensity curves from breast dynamic contrast enhanced MRI

**DOI:** 10.1186/s13046-015-0140-y

**Published:** 2015-03-04

**Authors:** Jiandong Yin, Jiawen Yang, Lu Han, Qiyong Guo, Wei Zhang

**Affiliations:** Department of Radiology, Shengjing Hospital of China Medical University, Shenyang, P.R. China

**Keywords:** Invasive ductal carcinoma, Receiver operating characteristic, Time intensity curve, Diagnostic performance, CAD, Semi-automatic method

## Abstract

**Background:**

Traditional subjective method for the analysis of time-intensity curves (TICs) from breast dynamic contrast enhanced MRI (DCE-MRI) presented a low specificity. Hence, a semi-automatic quantitative method was proposed and evaluated for distinguishing invasive ductal carcinomas from benign lesions.

**Materials and methods:**

In the traditional method, the lesion was extracted by placing a region of interest (ROI) manually. The mean curve of the TICs from the ROI was subjectively classified as one of three patterns. Only one quantitative parameter, the mean value of maximum slope of increase (MSI), was provided. In the new method, the lesion was identified semi-automatically, and the mean curve was classified quantitatively. Some additional parameters, the signal intensity slope (SI_slope_), initial percentage of enhancement (E_initial_), percentage of peak enhancement (E_peak_), early signal enhancement ratio (ESER), and second enhancement percentage (SEP) were derived from the mean curves as well as the lesion areas. Wilcoxon’s test and receiver operating characteristic (ROC) analyses were performed, and *P* < 0.05 was considered significant.

**Results:**

According to the TIC classification results, the accuracies were 59.16% for the traditional manual method and 76.05% for the new method (*P* < 0.05). For the mean MSI values from the manual method, the accuracy was 63.35%. For the mean TICs derived from the semi-automatic method, the accuracies were 77.47% for SI_slope_, 65.24% for MSI, 58.45% for E_initial_, 66.20% for E_peak_, 71.83% for ESER, and 54.93% for SEP, respectively. For the lesion regions identified by the semi-automatic method, the accuracies were 73.24%, 72.54%, 58.45%, 62.68%, 64.09%, and 55.64%, respectively.

**Conclusion:**

Compared with traditional subjective method, the semi-automatic quantitative method proposed in this study showed a higher performance, and should be used as a supplementary tool to aid radiologist's subjective interpretation.

## Background

Breast cancer is one of the most common malignancies in women worldwide and the second leading cause of cancer death among women [1-3]. Although numerous advances in prevention, surgical resection, and adjuvant chemoradiotherapy have led to a decline in the overall mortality due to breast cancer, the survival rates for patients with metastatic disease have not significantly improved. Consequently, the discovery of novel technique involved in the diagnosis of breast cancer is of great value.

Due to its three-dimensional nature, MRI has been considered as complementary to conventional mammography for the evaluation of suspicious breast lesions [4]. It can detect cancers missed by mammography or ultrasound in women who have dense breasts. Thus, the American Cancer Society has proposed that women with a lifetime breast cancer risk of 20% or greater should receive an MRI examination starting at age 20–30 years [5,6]. Dynamic contrast enhanced MRI (DCE-MRI) is one of the main imaging protocols that can provide a series of high spatial resolution images over time. The advantage of DCE-MRI over conventional imaging techniques (such as mammography and ultrasonography) is its ability to obtain and analyze both morphological and functional features corresponding to lesion characteristics [4,7]. For example, the time intensity curve (TIC) of signal from DCE-MRI has been frequently used clinically to characterize the biological and clinical aggressiveness of breast lesions [7-9]. A key characteristic of the TIC is the shape of the washout portion [10]. Generally, DCE-MRI has been qualitatively analyzed using commercially available software. First, a circular region of interest (ROI) was placed by a breast radiologist onto the parametric map reflecting the maximum slope of increase (MSI) to include the suspicious lesion. The mean TIC of signals within the ROI was calculated, and then was subjectively classified as persistently enhancing (type I), where the signal intensity continued to increase over time; plateau (type II), where the signal intensity did not change over time after its initial increase during the delayed phase; and washout (type III), where the signal intensity decreased after reaching the highest point of its initial increase during the delayed phase [10-16].

For conventional analysis of DCE-MRI based on the manual method, there are several disadvantages. First, the manual method is very time-consuming [17]. It is very important to minimize reading time in large hospitals like ours, which serve over 10,000 patients a day. Second, due to a dependence on the operator, the results from the manual method lack reproducibility for the same observer at different time points and for different observers at the same time point. Inter- and intra-observer variabilities have been considered a substantial limitation of the manual method [18-22]. Furthermore, inconsistent breast DCE-MRI interpretations could result in adverse effects on disease diagnoses and evaluations of treatments. Third, for the complete manual method, accurate results were adversely affected by partial volume effects when viable tumor tissue and necrosis were closely located [22,23]. Sometimes, it is really difficult to assess the lesion's margins [6]. Fourth, recently some studies have reported that although the conventional method resulted in higher sensitivity in the determination of breast lesions, its specificity was low or moderate [4,10,11,19,24-28]. Hence, in order to overcome the weaknesses of the conventional method for DCE-MRI analysis, it is necessary to develop a computer-aided approach to increase the reading speed of breast DCE-MRIs, to decrease the variability between inter- and intra-observers, and to reduce the partial volume effect, and to improve the diagnostic specificity.

In the present study, we described a novel approach for the analysis of signal TICs from breast DCE-MRIs. In this novel method, the breast lesion area was better identified using a semi-automatic segmentation algorithm. Compared with traditional method drawing the breast lesions manually, this algorithm might be able to reduce the influence of partial volume effects on the subsequent analysis. In addition, in order to avoid the limitations involved in completely subjective determination of TIC patterns with naked eyes according to their shape, the current method quantitatively classified the TICs as one of three patterns. In our opinion, relative to traditional subjective method, this can lead to more reproducible results. To interpret DCE-MRI better, a variety of computer-aided diagnosis (CAD) methods for the analysis of enhancement kinetics were developed, among which some focused on the measurement of hemodynamic parameters based on Tofts and Kermode models, and others concentrated on the analysis of TIC shape characteristics. In this study, more enhancement information was obtained by measuring a series of quantitative parameters, including signal intensity slope, initial percentage of enhancement, percentage of peak enhancement, early signal enhancement ratio, and second enhancement percentage. The DCE-MRI data could therefore be utilized more adequately. Because invasive ductal carcinoma (IDC) is the most common type of breast carcinoma (accounting for 70%-80%) [29,30], this type of malignant lesion was selected as the research subject in the present study. The prevalence of the investigated disease can make the study more meaningful and facilitate the case collection.

To the best of our knowledge, this is the first study comparing the traditional manual method and the novel method which has been currently proposed for the discrimination of breast IDCs and benign lesions. Overall, this comparison should be useful in the improvement of breast DCE-MRI diagnosis.

## Materials and methods

In this study, the performances of the conventional manual method and the currently proposed semi-automatic method were evaluated. The traditional method was performed based on commercially available software embedded in the dedicated workstation (FuncTool 9.4.05A, GE Healthcare, Milwaukee, WI, USA). The newly proposed method was performed using MATLAB software (version R2010b; The MathWorks, Inc., USA) developed by ourselves. The detailed process is described below.

### DCE-MRI acquisition and case collection

This study was approved by the ethics committee of Shengjing Hospital. Because this is a retrospective study, and all the cases used in this study were collected from the server of our PACS, written informed content from each patient was waived.

All breast DCE-MRI images were acquired using a 3.0 tesla scanner (Signa HDxt; GE Healthcare, USA), using a dedicated surface multichannel coil with the patient in the prone position. After axial localization, dynamic examination was performed using the VIBRANT-VX sequence with the following parameters: TR 7.42 ms, TE 4.25 ms, flip angle 15°, slice thickness 2.2 mm, spacing between slice 2.2 mm, inversion time 20 ms, image matrix 1024 × 1024, temporal acquisition 80 s, slice number 78. The three-dimensional scanning sequence was performed once before, and continuously eight times after intravenous injection of the contrast agent (0.5 mmol/ml, Gadodiamide, Omniscan,GE Healthcare; Magnevist, Bayer-Shering Pharmaceuticals). The contrast-enhanced study was performed with a bolus dose of 0.15 mmol per kilogram bodyweight, infused in the antecubital vein by a power injector at a rate of 4 ml/s. After that, an equal volume of saline flush succeeded at the same flow speed.

The breast DCE-MRI images acquired between January 2009 and August 2014 were read retrospectively by a breast-radiologist (13 years work experience in breast MRI). Although some patients underwent multiple breast DCE-MRI examinations before and after chemotherapeutic or surgical treatment, in the present study, only the images used for diagnostic purposes before treatment were collected for subsequent analyses. In order to facilitate the segmentation of breast lesions, all the lesions were mass-like and single (the lesions were either in the left or right breast). In addition, each breast lesion was verified as IDCs or benign lesions by biopsy or pathology after the DCE-MRI examination (time interval between MRI and histopathology examination was less than 5 days). As a result, 142 cases (all female; age range, 22–79 years; mean age, 53.5 years) were collected (71 benign cases and 71 IDCs). The detailed diagnoses of these benign lesions confirmed by pathology or biopsy are summarized in Table 1.

**Table 1:** **Detailed histopathological diagnoses and proportions of benign breast lesions**

**Lesion type**	**n**	**%**
High risk (complex sclerosing lesion, FEA, CCC with focal atypia)	5	7.04
Fibroadenoma, fibroadenomatous hyperplasia	33	46.48
Papilloma	4	5.63
DH, CCC, FCC, focal fibrosis, nodular sclerosing adenosis	16	22.54
Miscellaneous (chronic abscess, gynecomastia, fat necrosis, pseudoangiomatosis)	13	18.31

FEA = flat epithelial atypia, CCC = columnar cell changes, DH = ductal hyperplasia, FCC = fibrocystic changes.

### Conventional method for TIC analysis

**Figure 1:**
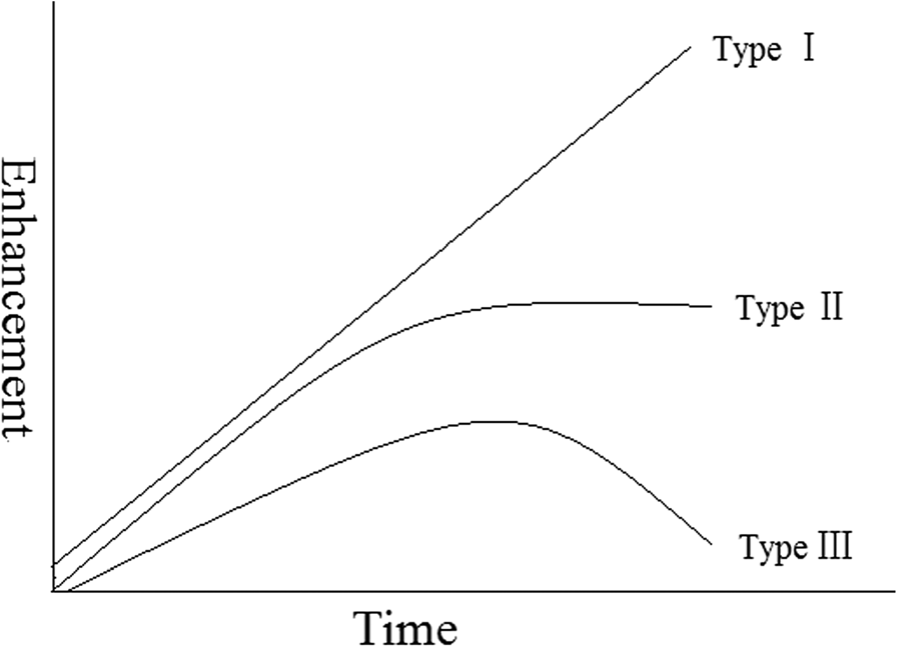
**Different patterns of signal TICs.** Type I is a progressive enhancement feature involving a continuous increase in signal intensity over time. Type II consists of a plateau pattern where there is an initial contrast-molecule uptake, followed by a unalterable phase. Type III shows a washout feature where there is an initial rapid uptake followed by reduction in signal intensity.

The selected images were transferred back to the workstation from the PACS server for interpretation using the dedicated software (FuncTool). Conventional methodology for the analysis of TIC from DCE-MRI was performed by an experienced breast-radiologist, who was blinded to the patient’s clinical information. Image subtraction of basal acquisition from the post-contrast dynamic images was performed to detect the suspicious lesions (enhanced areas). As suggested by Cheung et al., the third post-contrast subtracted image was used for better visualization of the lesion margin, and the slice with the maximum sized lesion was selected for subsequent analysis [13]. FuncTool produced a MSI map, and a circular ROI was manually placed onto the MSI map to cover the suspicious lesion. Then the TICs from the ROI were automatically averaged. Based upon the reader's subjective judgment, the mean curve was categorized as "continued signal intensity increase" (type I), "plateau" (type II), or "washout" (type III), as shown in Figure 1 [4,29,31]. The mean MSI value from the ROI was also automatically provided by the dedicated software.

### A novel method for TIC analysis

#### Semi-automatic segmentation of breast lesions

To eliminate the defects of manual extraction of breast lesions, the proposed method determined the lesion area semi-automatically. The segmentation procedure of breast lesions involved the following steps.

First, a ROI of arbitrary shape was drawn around the lesion;

Second, Ostu's algorithm was applied to the data from ROI [32], based on which pixels were divided into two parts, i.e. background and foreground;

Third, morphological erosion was applied to the binary image obtained above, and the size of structure element was 4 × 4;

Fourth, the post-eroded image was traversed to obtain the unique but largest eight-connected region;

Finally, morphological dilation was applied to the unique region with a structure element of the same size. The final region was considered as the target area corresponding to the lesion.

### Quantitative analysis of TIC

First, the TICs of signals in the target area were averaged. In order to classify the mean TIC quantitatively, the slope of signal intensity (SI_slope_) was measured using the following equation:

1 $$ \mathrm{S}{\mathrm{I}}_{\mathrm{slope}}=\left[\left(\mathrm{S}{\mathrm{I}}_{\mathrm{tail}}-\mathrm{S}{\mathrm{I}}_{\mathrm{mean}}\right)/\mathrm{S}{\mathrm{I}}_{\mathrm{mean}}\right]\times 100\% $$

where SI_tail_ was the value of the curve at the last time point, and SI_mean_ was the mean value of the first two post-contrast time points (i.e., the value at the time point of 120 S).The above mean curve was automatically designated typeIwhen the SI_slope_ value was +10% or larger, type II when the SI_slope_ value was between −10% to +10%, and type III when the SI_slope_ value was −10% or lower [7,17,33]. Meanwhile, the SI_slope_ corresponding to the TIC of each pixel was also calculated automatically, and the parametric map was obtained as well as the mean SI_slope_ value. Furthermore, the following additional quantitative parameters not provided by the existing dedicated software were also derived from the mean curve.

1) Maximum slope of increase:

2 $$ \mathrm{M}\mathrm{S}\mathrm{I}= \max \left(\mathrm{S}{\mathrm{I}}_{\mathrm{i}+1}-\mathrm{S}{\mathrm{I}}_{\mathrm{i}}\right) $$

where SI_i_ and SI_i + 1_ denoted the signal intensities of the former and the latter phases, respectively, with *i* ranging from 0 to 7.

2) Initial percentage of enhancement (E_initial_):

3 $$ {\mathrm{E}}_{\mathrm{initial}}=\left[\mathrm{S}{\mathrm{I}}_1-\mathrm{S}{\mathrm{I}}_0\right]/\mathrm{S}{\mathrm{I}}_0\times 100 $$

where SI_1_ and SI_0_ represented the signal intensities of the first post-contrast phase and the pre-contrast phase, respectively [34].

3) Percentage of peak enhancement (E_peak_):

4 $$ {\mathrm{E}}_{\mathrm{peak}}=\left(\mathrm{S}{\mathrm{I}}_{\mathrm{peak}}-\mathrm{S}{\mathrm{I}}_0\right)/\mathrm{S}{\mathrm{I}}_0\times 100 $$

where SI_peak_ represented the peak value of the contrast enhancement [7,34].

4) Early signal enhancement ratio (ESER): [35]

5 $$ \mathrm{ESER}=\left(\mathrm{S}{\mathrm{I}}_1-\mathrm{S}{\mathrm{I}}_0\right)/\left(\mathrm{S}{\mathrm{I}}_2-\mathrm{S}{\mathrm{I}}_0\right)\times 100 $$

where SI_2_ represented the second post-contrast phase.

5) Second enhancement percentage (SEP): [34]

6 $$ \mathrm{S}\mathrm{E}\mathrm{P}=\left(\mathrm{S}{\mathrm{I}}_2-\mathrm{S}{\mathrm{I}}_0\right)/\mathrm{S}{\mathrm{I}}_0\times 100 $$

In a similar manner, the above parameters for the target region were also calculated on a pixel-by-pixel basis, and the color-coded map corresponding to each type of quantitative parameter, as well as the mean value, were automatically obtained.

### Statistical analysis

For each type of quantitative parameter, receiver operating characteristic (ROC) analysis was performed using the statistical software MedCalc (version 14.10.20, http://www.medcalc.org/). The area under the ROC curve (AUC), as an index of diagnostic performance was provided automatically as well as the optimal threshold, based on which specificity, sensitivity, and accuracy were obtained.

For the classification of mean TIC, cases with washout or plateau curves (type II or III) were generally classified as malignant, with the remaining cases classified as benign [36,37]. Based on these assignments, the specificity, sensitivity, and accuracy were again respectively obtained [38]. The paired-samples Wilcoxon test was performed using SPSS software (version 16.0) for comparison between the manual method and the proposed method. The difference was considered significant with a *P* value less than 0.05.

### Ethical standards and patient consent

Ethical clearance for this study was obtained from the Ethics Committee at Shengjing Hospital, which is part of China Medical University. Because this is a retrospective study, and all the cases used in this study were collected from the server of our PACS, written informed content from each patient was waived.

## Results

**Figure 2:**
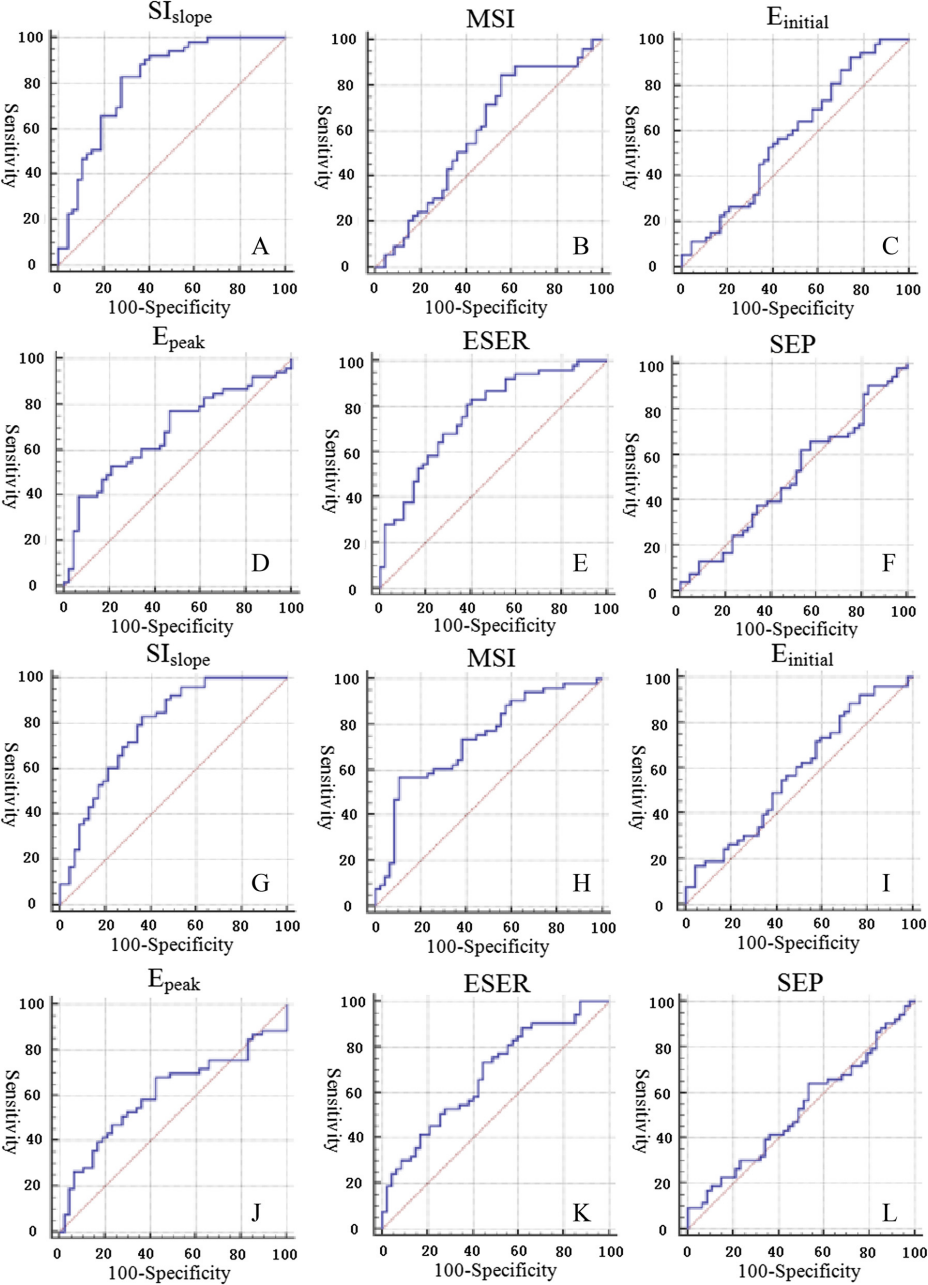
**Results of ROC analysis for the quantitative parameters. A-F** showed the results for the mean curve, and **G-L** showed the results for the target regions that were determined using the proposed method.

**Figure 3:**
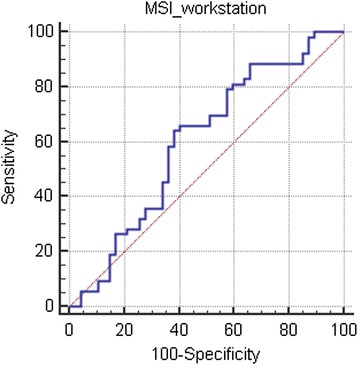
**ROC analysis for the mean MSI values derived from the manual method, with values of 0.601 for AUC, 0.0584 for SE, (0.498,0.698) for 95% CI, >884.4 for optimal cutoff value, 64.79% for sensitivity, 61.91% for specificity, and 63.35% for accuracy.**

For the quantitative parameters, the results of ROC analysis are shown in Figure 2, Table 2, and Table 3, and the results obtained using the conventional manual method are shown in Figure 3. The results of TIC classification are shown in Table 4. Statistical analysis showed that there was a significant difference in the TIC classification between the conventional subjective method and the new quantitative method (Z = −4.324, *P* < 0.05).

**Table 2:** **ROC analysis for the mean curve using the semi-automatic method**

	**SI**_**slope**_	**MSI**	**E**_**inital**_	**E**_**peak**_	**ESER**	**SEP**
AUC	0.813	0.595	0.578	0.676	0.765	0.501
SE	0.0443	0.0588	0.0583	0.0542	0.0475	0.0587
95% CI	(0.722,0.884)	(0.492,0.692)	(0.475,0.676)	(0.575,0.766)	(0.669,0.844)	(0.399,0.602)
Optimal cutoff	≤9.7039	>695.6076	>101.3672	≤202.115	>74.3415	>197.7642
Sensitivity	83.10%	84.51%	91.55%	39.43%	81.69%	63.38%
Specificity	71.83%	45.07%	25.35%	92.96%	61.97%	46.48%
Accuracy	77.47%	65.24%	58.45%	66.20%	71.83%	54.93%

SE = standard error, CI = confidence interval.

**Table 3:** **ROC analysis for the target region determined by the semi-automatic method**

	**SI**_**slope**_	**MSI**	**E**_**inital**_	**E**_**peak**_	**ESER**	**SEP**
AUC	0.784	0.737	0.578	0.606	0.679	0.523
SE	0.0465	0.0503	0.0579	0.0576	0.0534	0.0583
95% CI	(0.691,0.860)	(0.639,0.820)	(0.475,0.676)	(0.503,0.702)	(0.578,0.769)	(0.420,0.624)
Optimal cutoff	≤15.0926	>1294.9921	>112.8441	≤297.3396	>74.0187	>201.9853
Sensitivity	83.10%	56.34%	88.73%	67.61%	73.24%	64.79%
Specificity	63.38%	88.73%	28.17%	57.75%	54.93%	46.48%
Accuracy	73.24%	72.54%	58.45%	62.68%	64.09%	55.64%

**Table 4:** **Comparison of the TIC classification results obtained by the conventional and semi-automatic method**

	**Manual method**	**Semi-automatic method**
Sensitivity	85.92%	81.69%
Specificity	32.39%	70.42%
Accuracy	59.16%	76.05%

**Figure 4:**
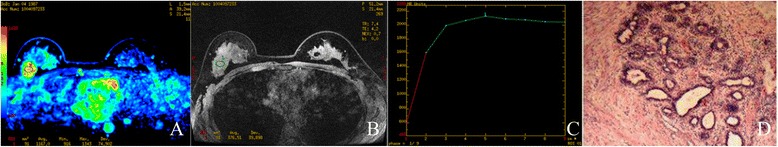
**Results based on the manual method for the randomly selected case. A-C** represent the MSI map, mask image (before injection of the contrast agent), and the mean curve of TICs from the manually drawn ROI, respectively. The mean curve was qualitatively designated as type II by the reader, and the mean MSI value was 1167.0. **D** is the pathological result showing breast adenosis (benign lesion).

**Figure 5:**
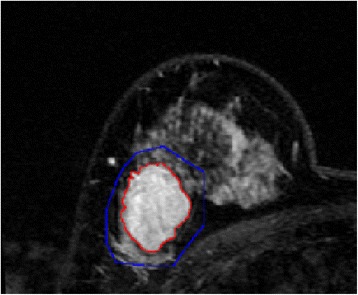
**Semi-automatic segmentation result of the breast lesion based on the proposed method for the randomly selected case (the colors were set to red for the lesion margin and blue for the ROI margin).** In order to facilitate the observation, this image partially enlarged.

**Figure 6:**
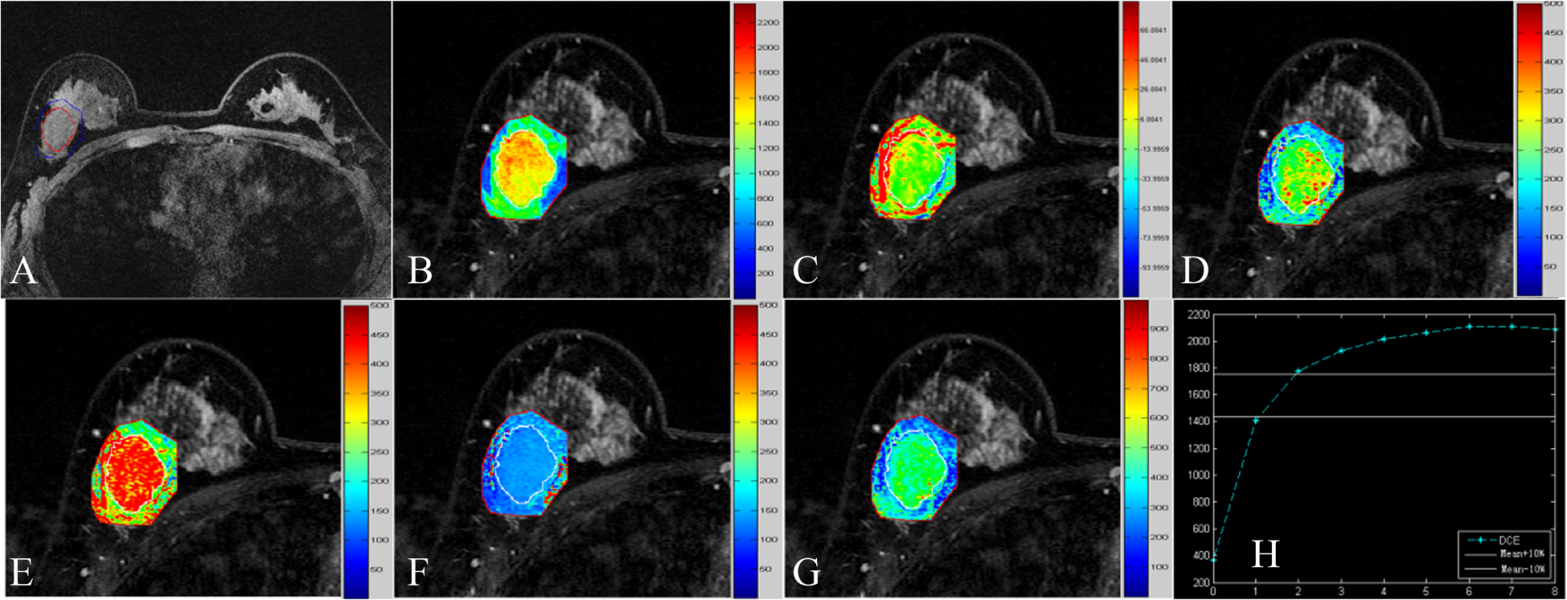
**Quantitative parametric map obtained using the proposed method. A-G** represent the mask image (before the injection of contrast agent) covered by the lesion margin, MSI map, SI_slope_ map, E_initial_ map, E_peak_ map, ESER map, and SEP map, respectively. Each image was partially enlarged. The mean values were 1827.479 for MSI, 41.002 for SI_slope_, 292.210 for E_initial_, 513.017 for E_peak_, 74.002 for ESER, and 394.252 for SEP. **H** is the mean curve of the TICs from the target region, which was quantitatively classified as type I. The above parameters for this curve were 1043.546, 30.731, 284.749, 475.910, 73.884, and 385.402, respectively.

A random case (45-years-old) was selected to illustrate the results obtained with the manual method (Figure 4), the procedure of lesion segmentation (Figure 5) and quantitative parametric maps (Figure 6).

## Discussion

As positron emission tomography, DCE-MRI can provide information about changes in vascularity, vascular permeability, and the relative volume of the extracellular space [28,29,39-41], but its cost is relatively low. It involves a serial acquisition of T1-weighted images before and after an intravenous injection of paramagnetic contrast agent (CA). As the CA enters into the tissue, the MRI signal intensity will be changed depending on the local distribution and concentration of CA. The MRI signal intensity will then return to the baseline value when the CA is transported out of the tissue. By analyzing the associated signal intensity time course, parameters related to physiological information can be obtained for each image pixel and region of interest [35]. Conventionally, the analysis of contrast-enhancement kinetics was achieved by placing ROIs manually. This manual method assumes accurate placement of the ROI in the most appropriate lesion area.

The semi-automatic method for TIC analysis proposed in the present study differs from the conventional manual method in several aspects, which result in potential advantages. First, the lesion determination was traditionally based on the MSI map, while in the proposed method it was based on the subtraction image between the post-contrast image and the pre-contrast image. Second, lesion extraction was previously based on a manual operation, but in the proposed study, it was completed using a semi-automatic segmentation procedure. Third, in the traditional method, TIC classification was subjectively determined by a reader’s visual inspection, but in the novel method, it was completely performed using a quantitative method. Finally, the dedicated workstation could only provide one quantitative parameter (MSI), but in the proposed method, several additional quantitative parameters related to the dynamic enhancement information were also measured.

Relative to the conventional method for TIC analysis of breast DCE-MRI, the new method proposed in the present study showed better performance in several aspects. For TIC classification, the conventional method resulted in relatively high sensitivity, but was also limited by low specificity, as reported in almost all the previous studies [4,10,11,19,24-28]. Indeed, low specificity decreased the overall accuracy. Relative to the diagnostic performance of the manual method, the sensitivity based on the semi-automatic method was slightly decreased, but specificity substantially increased, which resulted in better accuracy. For analysis of the mean TIC derived from the proposed method, the accuracy was greatest when using the quantitative parameter SI_slope_, and the sensitivity was greatest when using E_initial_, while the specificity was greatest using E_peak_. For analysis of the lesion region extracted by the semi-automatic method, again the greatest accuracy was obtained when using SI_slope_, but the specificity was not ideal (only 63.38%). The greatest specificity was obtained when using MSI, and the greatest sensitivity obtained when using E_initial_. For mean MSI values derived from the manual method, the accuracy only reached 63.35%. Thus it did not result in a diagnostic performance that could be better used in clinical practice.

The discrimination of benign and malignant lesions is an important basis for breast conserving therapy. Based on the results of the present study, we determined that the quantitative parameter SI_slope_ provided in the proposed method could be used to increase the specificity of breast DCE-MRI interpretation, and further improve diagnostic performance. Among the quantitative parameters, SI_slope_ was the best indicator for discrimination between IDCs and benign lesions. Traditionally, the optimal threshold value was set at +10% [17,18,33]. However in the present study, we found more accurate results if the value was set at +9.7%. In our opinion, this previously selected slope value might be only an empirical value, and the establishment of a new optimal cutoff value could play an important role in future interpretations of breast DCE-MRIs, resulting in the avoidance of unnecessary surgeries or biopsy for benign lesions. We anticipate the results of the present study being most useful for guiding future studies and for motivating other investigators to further conduct retrospective analysis of similar datasets.

Over the last years, a number of studies on CAD for differentiating benign from malignant breast tumors on DCE-MRI have been carried out [16,38,42-47]. Yang et al. developed a computer-aided detection scheme to measure a global contrast enhancement feature, and the sensitivity reached 91.3%, but the specificity was only 66% [16]. In the paper reported by Levman J [5], a semi-automatic lesion segmentation based on a supervised learning formulation was proposed for the distinction between malignant and benign breast lesions, and improved the AUC from 0.75 to 0.79 when compared with traditional enhancement threshold method. Some previous papers also reported the value of hemodynamic parameters in the differentiating benign from malignant breast lesions, and presented better diagnostic performance relative to conventional kinetic curve analysis [10,47]. In a highly innovative paper [44], both morphological feature and kinetic curve were analyzed quantitatively, and a so-called morpho-dynamic index (MDI) was proposed. Using the MDI cutoff value of 50%, the sensitivity was 96.5% combined with specificity of 75.5%. Compared with the above previous reports, the currently proposed method provided more quantitative parameters reflecting the enhancement information of breast lesions, and the maximum AUC (0.813) in combination with sensitivity of 83.10% and specificity of 71.83% was obtained based on SI_slope_ derived from the mean curve. In our opinion, the diagnostic accuracy might be higher if the morphological features were also analyzed.

It must be emphasized that there are several limitations regarding this study. First, the sample amount in this study was insufficient to obtain a definitive conclusion. If the case size was changed, both the optimal cutoff value and accuracy rate might be changed accordingly. Second, both the manual method and the semi-automatic method were performed only once, hence the inter- and intra-observer variabilities were not investigated [26], although we assumed that the semi-automatic method might offer greater reproducibility by virtue of its simplicity and quantification, especially when deployed across multiple sites in a large-scale clinical trial [33,48]. Third, in this study, we only analyzed the TICs from breast DCE-MRI, whereas the morphologic features of the lesions were not utilized for breast diagnosis, which could have further improved the diagnostic accuracy of breast DCE-MRI [17,36]. Finally, in order to facilitate the segmentation, only mass-like lesions were included in this study. If we had included lesions with various characteristic patterns, the results of the discrimination between IDCs and benign lesions might have been different.

## Conclusion

In conclusion, the semi-automatic method proposed in this study can be applied to DCE-MRI for the distinction between breast IDC and benign lesions. Compared with the traditional method, the new method improved the specificity and showed promise in the development of future CAD of breast DCE-MRI.

## Abbreviations

MRI: Magnetic Resonance Imaging
PACS: Picture Archiving and Communication System
TR: Repetition time
TE: Echo time
SPSS: Statistical product and service solutions
